# Scipio: Using protein sequences to determine the precise exon/intron structures of genes and their orthologs in closely related species

**DOI:** 10.1186/1471-2105-9-278

**Published:** 2008-06-13

**Authors:** Oliver Keller, Florian Odronitz, Mario Stanke, Martin Kollmar, Stephan Waack

**Affiliations:** 1 Universität Göttingen, Institut für Informatik, Lotzestr. 16-18, 37083 Göttingen, Germany; 2 Max-Planck-Institut für Biophysikalische Chemie, Abteilung NMR-basierte Strukturbiologie, Am Fassberg 11, 37077 Göttingen, Germany; 3 Universität Göttingen, Institut für Mikrobiologie und Genetik, Abteilung Bioinformatik, Goldschmidtstr. 1, 37077 Göttingen, Germany

## Abstract

**Background:**

For many types of analyses, data about gene structure and locations of non-coding regions of genes are required. Although a vast amount of genomic sequence data is available, precise annotation of genes is lacking behind. Finding the corresponding gene of a given protein sequence by means of conventional tools is error prone, and cannot be completed without manual inspection, which is time consuming and requires considerable experience.

**Results:**

Scipio is a tool based on the alignment program BLAT to determine the precise gene structure given a protein sequence and a genome sequence. It identifies intron-exon borders and splice sites and is able to cope with sequencing errors and genes spanning several contigs in genomes that have not yet been assembled to supercontigs or chromosomes. Instead of producing a set of hits with varying confidence, Scipio gives the user a coherent summary of locations on the genome that code for the query protein. The output contains information about discrepancies that may result from sequencing errors. Scipio has also successfully been used to find homologous genes in closely related species. Scipio was tested with 979 protein queries against 16 arthropod genomes (intra species search). For cross-species annotation, Scipio was used to annotate 40 genes from *Homo sapiens *in the primates *Pongo pygmaeus abelii *and *Callithrix jacchus*. The prediction quality of Scipio was tested in a comparative study against that of BLAT and the well established program Exonerate.

**Conclusion:**

Scipio is able to precisely map a protein query onto a genome. Even in cases when there are many sequencing errors, or when incomplete genome assemblies lead to hits that stretch across multiple target sequences, it very often provides the user with the correct determination of intron-exon borders and splice sites, showing an improved prediction accuracy compared to BLAT and Exonerate. Apart from being able to find genes in the genome that encode the query protein, Scipio can also be used to annotate genes in closely related species.

## Background

In the post-genome era, sequence data is the entry point for many studies. Often, it is essential to obtain the correct genomic DNA sequences of eukaryotic genes because of the information contained in non-coding regions. For example, the intron regions contain important sites for the regulation of gene transcription, like enhancers, repressors, and silencers [1]. Transcription initiator sequences are located upstream from the target gene [2]. The determination of the exon/intron structures of genes is also important in comparative genomic analyses like the identification of ancient exons [3].

Today, over 340 eukaryotic genome sequencing projects have resulted in genome assemblies [4]. For many of these project, genomic sequences of genes are only available by ab-initio gene predictions, if at all. However, it has been shown that automatically annotated sequences are often wrong because of sequencing and assembly errors, and mispredictions of exons and introns [5]. Correct protein sequences have in many cases been derived from manual annotation of the genes of interest or from full-length cDNAs. But experimentally obtained cDNA sequences often do not completely correspond to annotated genes, for example because previously undescribed alternative splice forms have been isolated. In many cases, it might also be interesting to look at the genes of evolutionary closely related species. If these species have not been annotated yet, it is, however, very time-consuming to identify and manually annotate the corresponding homologous genes.

Currently, a few programs are available for the retrieval of non-coding sequence. The Java application Retrieval of Regulative Regions (RRE) parses annotation and homology data from NCBI [6]. RRE requires local installation and local copies of the desired genome and annotation files. The web application of RRE only hosts a small number of eukaryotic genomes and annotation data only from NCBI. Recently, the non-coding sequences retrieval system (NCSRS) has been published [7] that has 16 genomes and annotation data from both NCBI and Ensembl [8]. In summary, both tools rely on annotation files provided by NCBI and Ensembl, with all possible errors, for only a few organisms. In addition, Ensembl and the UCSC browser [9] allow to search for genes and to recover any part of the gene of interest. When searching with descriptive terms, accession numbers, or other search terms the output is mainly based on results of gene prediction programs, often supported by evidence from cDNA or manual curation. Both web-interfaces also allow searching the genomes with any protein query with either BLAST or BLAT. However, the quality of the resulting gene structure is limited by these programs. There are also further species-specific genome pages that offer retrieving the genomic DNA corresponding to search terms. But there is no service offering the retrieval of the gene structures corresponding to protein queries of almost all sequenced and assembled eukaryotic genomes.

If transcript or protein sequences are available, the task of automatic identification of gene-related sequences can be accomplished by spliced alignment tools much more accurately and faster than, for example, by ab initio gene prediction. Splign [10] is a program that aligns a cDNA query determining the exact location of splice sites. GeneWise [11] uses protein queries. The most notable tools that can do both are Exonerate [12], partly based on the algorithm used in GeneWise, and BLAT [13]. The suitable variants of BLAST [14] (TBLASTN etc.) are probably still the most widely used tools for the task, though they are more useful for detecting weak homology and yield separate contiguous hits rather than a single complete spliced alignment. All approaches have in common a tradeoff between high speed that can only be reached using heuristics, and optimal accuracy that can only be achieved by exact algorithms that are prohibitively slow for many applications. Exonerate is very flexible as it implements a variety of models to choose from in a particular setting. BLAT is specialized on queries with high sequence similarity, and is considered the fastest tool for this task.

An accurate protein spliced alignment tool is particularly useful when a cDNA sequencing project precedes a genome sequencing project. In those cases, protein sequences can be constructed using the cDNA but the genomic location of the exons and introns is yet unknown. If the genome assemby is fragmented and genes are split onto different contigs, then any gene-finding or alignment method that considers each contig separately makes insufficient use of the available protein sequence information. Here, a spliced alignment should take contig-spanning introns and exons into account for maximal accuracy at the contig boundaries. Another application scenario for accurate protein spliced alignment is the problem of annotating a new assembly of an already annotated genome. Frequently, a complete re-annotation is time-consuming as it often requires different groups to run different gene finders and integrating all available evidence. Simply mapping the previously known protein sequences to the new genome is a fast and easy alternative, at least for those genes that can be mapped with 100% identity.

Some sequencing approaches sequence only filtered genomic regions that are enriched for genes like the methyl-filtrated sequencing or high-cot analysis used for many plants (e.g. maize and tobacco). In these cases the assembly will remain fragmented and many genes are split up onto different contigs, requiring that a spliced alignment tool takes this fragmentation into account.

Here, we present Scipio, a tool for the retrieval of the genome sequence corresponding to a protein query. It uses BLAT to perform a spliced alignment that results in a list of candidate hits, which are then refined, filtered and assembled to produce a prediction as accurate as possible. No annotation data is required, and genes are identified correctly even if they span several genome contigs, and contain mismatches and frameshifts. With these capabilities, Scipio is not only able to correctly identify the gene in the genome corresponding to the protein query but also to identify homologous genes in the genomes of closely related organisms. Moreover, it provides a comprehensive output, both machine- and human-readable, already containing the genomic sequences searched for.

## Implementation

In most instances, the task of determining the gene structure of a query protein within a DNA target sequence is a special case of the search for a spliced alignment. Since several tools performing this task have already been available for a long time, writing another one would mean reinventing the wheel. Our choice was to depend on a BLAT search.

However, in the example of BLAT, when performing a search for the protein in the translated DNA, the output does not coincide with the exon structure of a single gene. Usually, multiple hits are found for each query, varying in accuracy, and exon boundaries are given only on amino acid level, missing those codons that are split by introns. Hence, manual processing was still needed until now in the majority of cases to determine the exact location of the query. In cases where the genomic sequence is in an early stage of the assembly process, several parts of one particular gene are often found on different target sequences (contigs), making this task very tedious and time consuming.

### The Scipio script

**Figure 1:**
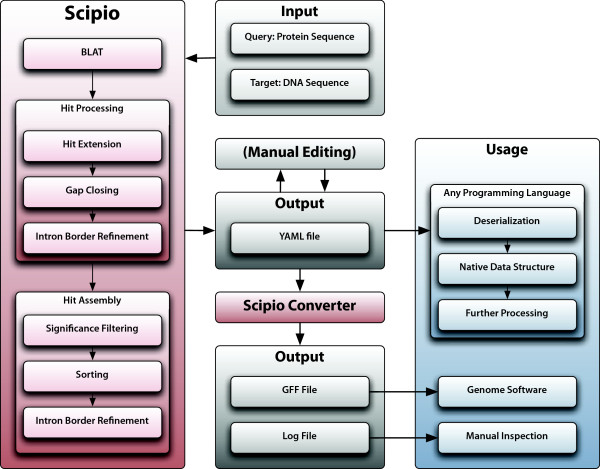
**The Scipio workflow**. This diagram depicts the data flow of a Scipio run. Scipio needs two FASTA files as input, one containing the protein query and one containing the genome sequence. Scipio starts BLAT and processes the BLAT results in a series of steps, successively refining and assembling the hits. Scipio's output is a YAML file, which can further be converted into a GFF file or a log file. YAML files can also be manually edited and read by a parser of which many exist for all modern programming languages. The resulting data structure can then be further processed.

We designed the perl script Scipio to automate this process and output the results in both human- and machine-readable output formats [see Additional file 1]. The summary of the process is depicted in the diagram in Fig. 1. We chose to run BLAT to provide us with the spliced alignments because it is specialized for the case of high sequence identity, which is obviously the case when locating genes of the same species (where mismatches are mainly due to sequencing errors), but it turned out to be very applicable also for the case of closely related species.

#### Stage one: hit refinement

After running BLAT, Scipio processes the query protein and target DNA sequences, and the file containing the BLAT hits. In the first stage, each hit is then "refined" by a number of steps. A BLAT hit is a collection of consecutive matchings of the protein sequence aligned to the translated DNA. We do not want to include hits with low quality. Therefore, everything with an accuracy below a given threshold is discarded at this stage. The refinement then consists of the following steps:

• Unaligned parts of the target sequence between the matchings that form a BLAT hit are analysed. A hit is only likely to be considered if they consist of a longer piece of DNA corresponding to at most one residue of the query, so they can be regarded as introns. Scipio tries to determine the exact location of the splice site by looking for a known splice site pattern (see below). This way, codons that are split by an intron, and are only joined after splicing, can be revealed. In cases where all residues are aligned by BLAT but a splice site pattern is missing, Scipio tries to improve the prediction by shifting the splice sites in single nucleotide steps. If an exact location can not be found, a heuristic is used to determine a trade-off between the number of additional mismatches and the presence of the splice site pattern.

• In addition, two more types of unaligned target sequence are distinguished: First, actual gaps with significant parts of the query sequence unaligned (mostly due to low coverage of sequencing resulting in gaps between contigs represented by contiguous N's in supercontigs/chromosomes). Second, short gaps resulting from sequencing and assembly errors leading to additional or missing bases or codons, with or without a frameshift. Additional DNA in this case is not interpreted as intron an but as a sequence shift of the query against the target.

• Scipio tries to locate very short exons where the BLAT hit misses parts of the query sequence. This is done by simple pattern matching. Thus only pieces with full identity are added. Terminal exons are added only when an intact splice site is found.

The filtering during the first stage ensures that nothing will be shown that cannot be regarded as a good match. If no hit is left after filtering, Scipio simply considers the gene non-existent in the target sequence, and no further processing is done.

#### Stage two: hit filtering and assembly

All BLAT hits that survive the first stage are subsequently filtered in the second stage to determine those that form the gene corresponding to the protein query. If only complete chromosomes were considered, one could expect a single optimal BLAT hit coinciding with that gene; however, in cases without a complete assembly, partial hits on different targets need to be taken into account.

First, all hits are sorted by a score proportional to the number of matches, with a penalty subtracted for each mismatch. Second, all incompatible hits are discarded in the order just determined. Hits are incompatible if their queries overlap but their targets do not. An exception is the complete identity on DNA level at the ends of two contigs. This could result from an incomplete assembly, and the possibility of an overlap is taken into account. At the end of this step, we come out with a small number (usually just one) of non-overlapping hits forming the best gene candidate.

The final part of stage two is another refinement step: by assembling multiple hits, sequence parts may have been identified as parts of an intron that is split on different targets, the first half at the end of one target, the second at the beginning of the next. After the assembly Scipio uses the same method as in stage one to determine the exact splice site locations.

### Output

The output contains target names, and location coordinates (genomic and protein) of all features: introns, exons, and gaps; exons can have sub-features: sequence shifts, mismatches, or undetermined positions. In addition, it contains the genomic DNA for all regions (including up- and downstream of the hit) and the translation of the coding sequence.

For the output format we defined two essential requirements: Human readability and machine readability. We chose YAML as it is a format that is complex enough to express our data structures and at the same time simple enough to be human readable and editable. YAML can be parsed easily and has numerous bindings to any modern programming language. The resulting native data structures can be used to further process the data generated by Scipio.

### Conversion tools

Scipio provides two tools to convert YAML files:

• yaml2log: Converts YAML files into an easily readable log file with summary information about the results and clearly arranged sequence alignments.

• yaml2gff: Converts YAML files into GFF Format which can be read by a wide range of genome-related software packages.

## Results and discussion

In many biological studies, protein sequences have been obtained by isolating mRNAs and sequencing the reverse-transcribed cDNAs. Also, large-scale cDNA sequencing projects resulted in thousands of supposed-to-be full-length cDNA sequences for some eukaryotes [15,16]. Protein sequences might have also been obtained by manual annotation. Sets of genomic DNA sequences of genes exist for some annotation projects. However, for many eukaryotic sequencing projects, the annotation process is lacking years behind the sequencing and assembly. In addition, experimentally obtained cDNA sequences often differ from annotated sequences because new alternatively spliced forms have been isolated. Therefore, for subsequent studies it might be useful or crucial to obtain the genomic DNA and the gene structure corresponding to the protein of interest.

Scipio has been designed for this task, and based on its differentiated processing capabilities it is able to cope with genes spanning multiple contigs as well as various kinds of sequencing and assembly errors. Scipio has been developed for the correct identification of eukaryotic genes. It can also be used for bacterial and archaeal genes although these genes are easily identified manually based on their simple single-exon structure. Depending on the similarity of the protein sequences, Scipio is also often able to correctly identify homologous genes in closely related organisms.

We have implemented the following features:

A. If the query is distributed on several targets, the target contigs will be assembled guided by the alignments to the query. Untranslated regions from the last exon on a contig to the contig end and from the beginning of the next contig to the next exon are regarded as intronic. Scipio is also able to resolve overlapping contig ends if they consist of coding sequence, hence contributing to an improvement of the assembly.

**Figure 2:**
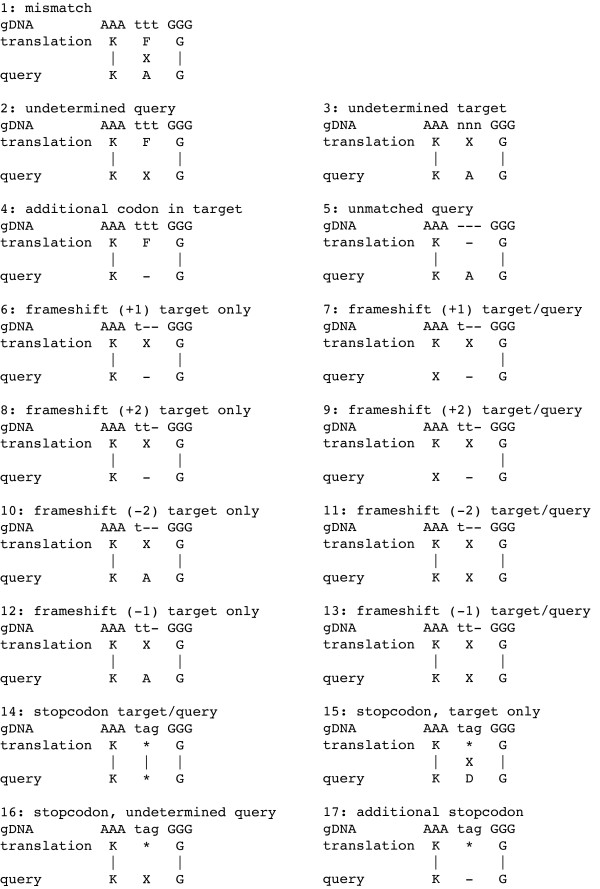
**Types of discrepancies**. This chart lists all types of discrepancies between the protein query and target translation/DNA that are known to Scipio. The identifiers as written into the log files are given.

B. The yaml2log script identifies cases from a list of alignment discrepancies and mismatches between query and target sequence that can result from sequencing/assembly errors (Figure 2). The simplest case is that amino acids differ (cases 1 to 3), or that they are missing in either the target or the query (cases 4 and 5). Sequencing/assembly errors may lead to additional or missing bases. These frameshifts are represented by an X in the translation corresponding to one or two nucleotides. The query sequence might have either been obtained from cDNA sources thus leading to a mismatch between query and translated target (cases 6, 8, 10, and 12), or the sequencing errors might have already been interpreted represented by an X in the query (cases 7, 9, 11, and 13). The target sequence might also contain in-frame stop codons (cases 14 to 17). These can be the result of sequencing errors or real stop codons as they appear in pseudogenes. In all these cases, the stop codon is shown as an asterisk ('*') in the translation.

C. Scipio interprets splice site patterns to determine intron locations. Exons borders are chosen so that the splice sites belong to one of the following classes, in decreasing priority: GT-AG, GC-AG, AT-AC, GA-AG, GG-AG. In cases where the translation of the adjacent intronic sequence was identical to the query, it was necessary to shift the intron location predicted by BLAT by several codons to determine the splice site location.

D. Scipio searches for stop codons at the end of genes. This helps evaluating the completeness of the query sequence.

E. Scipio tries to locate very short exons that are not recognized by BLAT. These short exons might either appear in-between longer exons or at the ends of the gene. For example, very often genes start with an N-terminal methionine that is the only translated codon in the first exon. Scipio locates N-terminal methionines only if matching splice sites are found.

### Insect genomes

**Figure 3:**
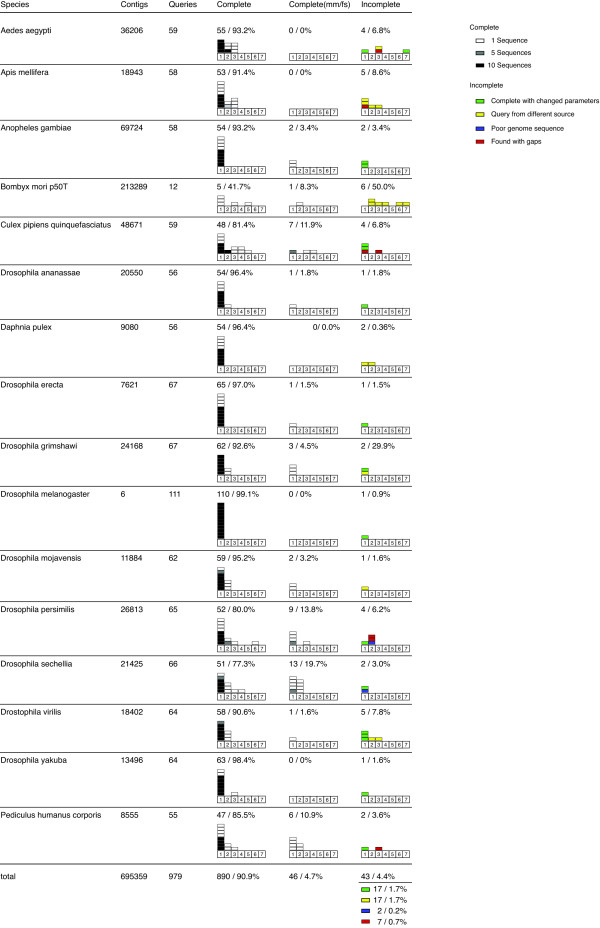
**In-Species Performance**. This chart shows Scipio's performance when searching in-species. The charts shows histograms depicting how many sequences where found on a particular number of contigs in the genome. Black rectangles represent ten, grey ones five and white ones single sequences. 'Complete' means the queries where found without discrepancies. 'Complete (mm/fs)' means that Scipio found the complete gene without gaps but with discrepancies like mismatches or framshifts. 'Incomplete' means that Scipio could not determine the complete gene structure with standard parameters.

To develop and test Scipio we used a test set of 16 arthropod genomes and a set of 979 proteins (Figure 3). The genome sequences (the newest collections of contigs as submitted to NCBI) differ in quality and completeness and are thus representative for straight-forward and for difficult identifications of the genes.

*Drosophila melanogaster *is an example of a perfect genome sequence with all reads assembled to chromosomes and almost all gaps closed. *Bombyx mori p50T *was used as example for a very preliminary assembly with many short contigs. The other genome sequences represent all stages in-between these extreme cases. For example, the genomes of *Drosophila persimilis *and *Drosophila sechellia *are quite complete which is visible from their number of contigs, but they have a low sequence coverage and/or contain many sequencing errors leading to high numbers of mismatches and frameshifts in the identified genes (Figure 3). In total, almost all query sequences have been identified correctly by Scipio (90.9 %), although many are spread on several target contigs (e.g. see *Aedes aegyptii *and *Culex pipiens*). 4.7 % of the genes have correctly been identified but the target DNA sequence contains sequencing or assembly errors. Another 1.7 % has not completely been found with the standard BLAT settings (BLAT tilesize of 5) because these genes contain very short exons. After changing the BLAT tilesize to 3 or 4 these genes have also completely been identified. Further 1.7 % of the genes could not be identified correctly, because the query sequence has been derived from manual annotation thus having incorporated EST data, data from other genome assemblies (e.g. newer data from the sequencing centers), or errors in the manual annotation process. E.g., the *Bombyx mori p50T *genome data is very incomplete but a lot of EST data is available. Thus, the query protein sequences have been built to a large part on these EST data. EST data has also been used to close gaps in the *Apis mellifera *and *Drosophila virilis *genomes. Errors resulting from this process are not due to problems in the implementation of Scipio. Two sequences (0.2 %) could not be identified correctly, because the genome sequences shows an large number of sequencing errors resulting in a succession of frame shifts in this particular region. The query protein sequences have correctly been identified based on EST data. The remaining 7 sequences (0.7 %) contain very long overlapping regions due to problems in the genome assemblies. Currently, Scipio handles overlapping hits by choosing the one with the higher overall score, in some cases discarding the one with fewer mismatches in the overlap region. The other cases in which Scipio did not resolve the complete gene structure are those, where a frame shift exists very close to an intron border. BLAT does not include the stretches past the frameshifts since they are smaller than the tile size used for searching. Scipio was not able to place the missing residues between the exons.

### Cross species search

To test the ability of Scipio to correctly predict orthologous genes in closely related organisms we have annotated the myosins in the recently assembled primates *Pongo pygmaeus abelii *and *Callithrix jacchus *[17]. As a query, we used the 40 manually annotated myosins from *Homo sapiens *[5], which can be obtained from CyMoBase [18,19]. Although the genome assembly is not complete and most of the sequencing/assembly errors described above have been seen, Scipio correctly predicted and identified most orthologs of the human myosins in the two primates and located all parts of the genes if they were distributed on several target contigs, It also correctly identified two rare splice sites (AT-AC and GG-AG) that are specific for vertebrate sequences in two myosin classes. In the tails of the class-15 and class-35 myosins very small and divergent gaps had to be filled manually.

**Figure 4:**
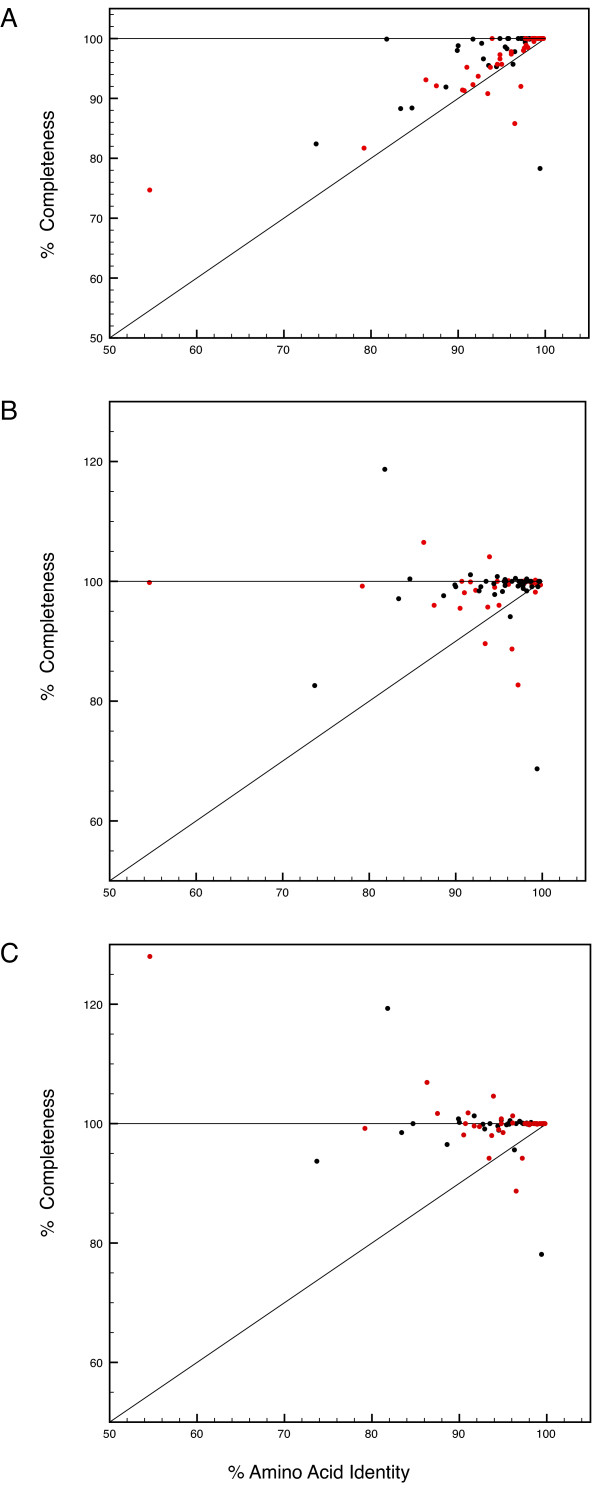
**Cross-Species Performance**. This chart shows Scipio's performance when searching cross-species. The charts show the dependency of the completeness of the gene reconstruction on the identity of the protein sequences. Red dots show searches with human sequences against the genome of *Pongo pygmaeus abelii*, black dots show searches with human sequences against the genome of *Callithrix jacchus*. A: Completeness compared to the query sequence. B: Completeness compared to the manually annotated sequence. A BLAT tile size of 7 was used. C: As in B, but with tile size 5.

Figure 4A, shows the completeness compared to the sequence of the human ortholog and demonstrates the incompleteness of the genome sequences of *Pongo pygmaeus abelii *and *Callithrix jacchus*. Figure 4B and 4C show how gene completeness as determined by manual annotation depends on sequence identity. Exceptionally low values are largely due to gaps in the genome sequence (see Figure 4A), values exceeding 100% are due to stretches in homologous genes that were found by Scipio. Comparison between Figure 4B and 4C illustrates how a more thorough search can improve the recognition accuracy of 'difficult' genes (e.g. genes with very short exons) but at the same adds false positive exons to genes.

### Comparison with existing tools

To assess the improvement on prediction quality that Scipio achieves, a comparative study was carried out with a selection of genomes varying in size and quality of assembly (Table 1). Running Scipio, standalone BLAT, and Exonerate, a number of myosin and kinesin queries were searched for in these genomic sequences.

**Table 1:** Sequences tested in comparative analysis.

Species	typ. target size (kbp)	# of target sequences	# of query sequences
*Bombyx mori p50T*	2.8	213 289	8
*Drosophila simulans*	9.4	31 198	37
*Bombyx mori str. Dazao*	14	66 482	32
*Nasonia vitripennis *(contigs)	27	26 605	35
*Nasonia vitripennis *(scaffolds)	1 827	6 181	35
*Drosophila sechellia*	70	21 425	38
*Aedes aegypti*	113	36 206	40
*Daphnia pulex*	825	9 080	42
*Anopheles gambiae*	6 969	69 724	37
*Drosophila melanogaster*	21 575	13	38
*Aspergillus niger*	2 409	143	15
*Homo sapiens*	153 287	24	40
*Macaca mulatta*	150 724	22	40

A list of 9 insect, one fungi, and two primate genomes, searched for kinesin and myosin genes to compare the performance of Scipio with that of BLAT and Exonerate. Genomic target sequences were taken from different stages of assembly, as can be seen from the different typical target sizes (*D. melanogaster*, *H. sapiens *and *M. mulatta *were given as sets of complete chromosomes; of the genome of *N. vitripennis*, two versions were compared; the genome of *A. gambiae *was given partly in chromosomes, partly in small contigs). The protein query sequences were taken from the same species as the genome.

Table 2 shows the accuracy on amino acid level. The improvement over BLAT is mainly due to the identification of split codons at intron boundaries. BLAT is only able to align unbroken codons, but will predict split codons as (false) matches whenever the splice site matches the query sequence. By the postprocessing, Scipio was able to reduce the number of residues missed by BLAT (including false positives) from 2.16 % to 1.67 %. Depending on the quality of the genomic sequences (some of which contained frameshifts, undetermined nucleotides, or even lacked larger parts), this is already the best that can be achieved theoretically; in the example of *Drosophila melanogaster *where complete chromosomes were available, 100 % of the residues could be matched.

**Table 2:** Percentage of residues left unmatched.

		BLAT	Exnrt.	BLAT	Exnrt.
			
Species	Scipio (automatic assembling)	with manual assembling		without assembling	
*Bombyx mori p50T*	15.03	15.41	17.03	43.61	43.64
*Drosophila simulans*	7.11	7.44	8.14	31.83	30.33
*Bombyx mori str. Dazao*	7.93	8.43	7.86	36.09	36.38
*Nasonia vitripennis *(contigs)	0.20	0.60	0.45	10.17	9.38
*Nasonia vitripennis *(scaffolds)	1.11	1.50	1.38	3.70	3.89
*Drosophila sechellia*	0.11	0.45	0.95	9.05	10.14
*Aedes aegypti*	0.73	1.01	0.21	10.43	10.22
*Daphnia pulex*	1.26	1.84	1.33	1.84	1.88
*Anopheles gambiae*	0.02	0.31	0.26	0.31	0.63
*Drosophila melanogaster*	0.00	0.32	0.05	0.32	0.05
*Aspergillus niger*	0.01	0.18	0.08	0.18	2.63
*Homo sapiens*	0.06	0.92	0.10	0.92	0.10
*Macaca mulatta*	2.29	3.09	2.31	3.09	7.82

total	1.67	2.16	1.87	8.24	8.71

The percentage of residues of the query sequences that the compared tools failed to recover from the target sequence. To gain comparable results, the hits proposed by BLAT and Exonerate were assembled together manually: a collection of best-scoring non-overlapping hits was chosen for each query. The last two columns show the results if only the best-scoring hit for each query was used.

Exonerate is able to find introns with exact boundaries. Hence, its accuracy is somewhat higher than BLAT's. However, in our setting, Exonerate had running times of about three to ten times longer than Scipio. Scipio adds less than ten percent to BLAT's running time.

It is important to note that manual postprocessing of BLAT's and Exonerate's results was used to gain comparable values: while BLAT and Exonerate come out with a collection of possibly overlapping hits with strongly varying accuracy, Scipio is able to filter and assemble non-overlapping partial hits on multiple targets together to a single prediction for each query sequence. This additional feature of Scipio had to be emulated by manually choosing a collection of best-scoring hits without large overlaps, and adding up the numbers of matches found, from the outputs of the two other programs.

In the case of a genome in a very early stage of assembly (here: the silkworm *Bombyx mori*), this assembling of hits increased the number of matching residues found to 92 % from 64 % which would be found taking only a single hit for each query. In the genome of *Macaca mulatta*, although target sequences were complete chromosomes, Exonerate still failed sometimes to return a single hit comprising the entire query sequence, and hence assembling of partial hits increased the number of residues found here, too. Manual inspection showed that Scipio produced false positives in rare cases when it rather had declared the gene not present in the target sequences. 248 residues (equaling 2 % of the missing residues, or 0.04 % of all) accounted for false exons.

The results on sequence level (Table 3) show strikingly how much the workload for manual postprocessing is reduced by Scipio, given that every sequence not matched completely will have to be looked at again, and alignments marked as completed (without mismatches, missing codons, sequence shifts or doubtful splice site patterns) will not.

**Table 3:** Percentage of perfectly aligned queries.

		BLAT	Exnrt.	BLAT	Exnrt.
			
Species	Scipio (automatic assembling)	with manual assembling		without assembling	
*Bombyx mori p50T*	25.0	0.0	25.0	0.0	12.5
*Drosophila simulans*	37.8	2.7	21.6	2.7	16.2
*Bombyx mori str. Dazao*	15.6	3.1	12.5	0.0	9.4
*Nasonia vitripennis *(contigs)	71.4	11.4	57.1	11.4	54.3
*Nasonia vitripennis *(scaffolds)	68.6	11.4	65.7	11.4	65.7
*Drosophila sechellia*	63.2	7.9	55.3	2.6	55.3
*Aedes aegypti*	87.5	7.5	52.5	7.5	50.0
*Daphnia pulex*	88.1	0.0	76.2	0.0	76.2
*Anopheles gambiae*	94.6	8.1	73.0	8.1	73.0
*Drosophila melanogaster*	100.0	10.5	86.8	10.5	86.8
*Aspergillus niger*	86.7	6.7	73.3	6.7	73.3
*Homo sapiens*	62.5	0.0	50.0	0.0	50.0
*Macaca mulatta*	12.5	0.0	10.0	0.0	10.0

total	64.5	5.5	51.7	4.8	50.3

The number of query sequences that were predicted by the programs exactly at the correct location, with 100 % matching residues, without frameshifts or false positives. This figure reveals the amount of workload needed for manual postprocessing of the hits.

While BLAT was unable to yield a complete prediction including the correct splice site locations in 95 % of the query sequences, Exonerate, by modeling introns, did better but would still fail to give a complete prediction in about half of the queries, even when partial hits were combined manually. The reason why assembling hits does not improve the number of completed queries is that in cases when a genomic sequence (e.g., a contig) ends in the middle of an intron, Exonerate will not use the intron model. Instead, at the cost of mismatches, the alignment is extended into the intron, yielding false boundaries and consequently, a false prediction.

In the case of *D. melanogaster*, where Scipio completely recovers all 38 query sequences, Exonerate misses five short exons; while these account only for 0.05 % of all residues, it reduces its rate of success on sequence level to 33 of 38, or 86.8 %.

These results show that Scipio, apart from providing a more detailed and well arranged output, can improve the prediction rate to 100 % by searching for short exons. In the case of fragmented genomes, the feature of hit assembly significantly improves the chances of retrieving the complete genomic sequence belonging to a protein query.

### Future plans

The primary aim of the upcoming version of Scipio is to eliminate false positive predictions and to close more gaps still left in the prediction.

Eukaryotic genes contain far more information than is encoded in the sequence of one expressed protein. Most of this information is contained in the untranslated regions. Therefore, our future developments will focus on analyzing the untranslated regions to provide the user with additional gene-related information. Thus, Scipio will be developed to identify untranslated exons and, in addition, to determine mutually exclusive exons, and other alternatively spliced exons.

A web interface for Scipio is currently under development to address a wider audience and to make Scipio more user-friendly.

## Conclusion

Scipio is a tool for the determination of gene structure and annotation of genes for a given protein sequence. Based on the widely used program BLAT, it performs exhaustive processing to ensure the best possible mapping of the protein onto the genome. By the ability of assembling partial hits ranging over multiple target sequences, Scipio goes beyond the scope of present spliced alignment tools and presents the user with a coherent set of matches that are often accurate to the level of single bases. Having a certain level of tolerance, Scipio can handle mismatches and frameshifts that often result from sequencing errors in genomes and cDNA. The same tolerance can be used to track down homologous genes in closely related species, allowing for cross-species annotation.

## Availability and requirements

Project name: Scipio

Project home page: 

Operating system: Platform independent

Programming language: Perl

Software requirements: Installation of BLAT and BioPerl.

Hardware requirements: BLAT may demand several times the genome size in RAM. If the RAM size is limiting, the most reasonable way is to split the genome and run Scipio against the split files.

License: Scipio may be obtained upon request and used under a Creative Commons License.

Any restrictions to use by non-academics: Using Scipio by non-academics requires permission.

## Abbreviations

BLAT: BLAST like alignment tool; YAML: YAML ain't markup language.

## Authors' contributions

OK, FO and MK set the requirements for the system, performed testing, and wrote the manuscript. MK, MS and SW initiated the project, OK wrote software. FO and MK carried out the analysis of the insect genomes and the cross-species searches, OK the comparative tests. All authors read and approved the final version of the manuscript.

## Supplementary Material

Additional file 1 Source code of the software, two Scipio output conversion tools, and a manual on the usage of the Scipio script. Click here for file
