# Supplementary material for: Scipio: Using protein sequences to determine the precise exon/intron structures of genes and their orthologs in closely related species
Source: BMC Bioinformatics. 2008 Jun 13;9:278. doi: 10.1186/1471-2105-9-278 (PMC2442105; doi:10.1186/1471-2105-9-278)
Supplement: Additional file 1 — Source code of the software, two Scipio output conversion tools, and a manual on the usage of the Scipio script. [file 1471-2105-9-278-S1.zip › usage.html]

## How to use Scipio (version 1.0)

#### Requirements

- Bioperl (download from  bioperl.org )
- YAML Perl module (download from  CPAN)
- BLAT binary (see instructions at  UCSC)
- Genome of choice in fasta format (you can search diArk for sequenced eukaryotic genomes)

#### What the Scipio script does

The intention of the script is to locate protein query sequences in DNA targets.

BLAT does most of the computational work, however, there is some important postprocessing that is performed by
the script. This includes the following tasks:

- BLAT does not try to align codons that are split by introns. The script searches for missing codons, preferring those that are split at splice sites, and adds the nucleotides to the corresponding exons.
- For each query sequence, most BLAT hits are discarded and only
  (the) one best hit is displayed. In our case, we are only interested
  in nearly 100% sequence identity.
- We are prepared for the case of genes that have partial hits on
  multiple targets. First, all BLAT hits are collected, and sorted by score. Then non-overlapping
  hits are taken to form a collection of hits of the same query.
  (This is very useful if the target file is from an early stage of
  assembly, when gene locations are likely to exceed contig boundaries.)
- Frameshifts that usually cause BLAT to split an exon into multiple separate matches, are joined back into a single match (this can optionally be disabled).
- Retrieve all the corresponding sequences and group them together with the BLAT results to form the output described below.

#### Usage

| scipio.pl [*〈options〉*]  *〈target〉*  *〈query〉* | | |
|
|  | 〈target〉  is a DNA file |
|  | 〈query〉  is a protein file |
|  | both in FASTA format |
|
| Options: | --blat\_output=*〈filename〉* | name of BLAT output file (.psl); if not specified, defaults to the newest psl file in the working directory, or to  Scipio*〈nnnn〉*\_blat.psl if none exists |
|  | --force\_new | run BLAT even if output file already exists (the default is never to run BLAT if the specified or default psl file would be overwritten) |
|  | --verbose | show verbose information (including progress) |
|  | --min\_score=*〈value〉* | minimal score of the best partial hit for a query |
|  | --min\_identity=*〈value〉* | minimal identity in any hit (default is 0.9) |
|  | --max\_mismatch=*〈value〉* | maximal number of mismatches in any hit (0 means any number, the default) |
|  | --split\_on\_fs | do not join matchings separated by frameshifts (the default is to consider them a single exon) |
|  | --region\_size=*〈value〉* | size of shown upstream/downstream sequence parts (defaults to 1000, maximum is 75000) |
|  | --force\_score=*〈value〉* | specify a score (between 0 and 1) that forces a hit to be shown even if it is contradicting a better one   (these hits are not assembled together and shown as *〈queryname〉*\_(1), etc.) |
|  | --partial\_target | accept BLAT output files containing hits referring to nonexistent target sequences |
|  | --show=*〈list〉* | which keys are to be shown in YAML output (details see below) |
|  | --show\_intron=*〈list〉* |
|  | --show\_exon=*〈list〉* |
|  | --show\_gap=*〈list〉* |
|  | --hide\_undef | hide keys with undefined or empty list values |
|  | --hide\_defaults | hide some keys if they have a default value |
|
| Options passed to BLAT (ignored when BLAT is not run): | | |
|  | --blat\_bin=*〈name〉* | name of BLAT executable, defaults to "blat" |
|  | --blat\_params=*〈params〉* | parameters passed to BLAT |
|  | --blat\_tilesize=*〈..〉* | (see BLAT usage information) |
|  | --blat\_score=〈..〉 |
|  | --blat\_identity=〈..〉 |

  

#### Output overview

The script produces an output in YAML format. Additional scripts are supplied that transform the YAML output into other formats (e.g. GFF3). The output is organized as follows:

For each query, a list of the matched parts is given:

*〈query 1〉*:

- *〈1st BLAT hit〉*

- *〈2nd BLAT hit〉*

...

*〈query 2〉*:

- *〈1st BLAT hit〉*

etc.

Usually, there is only one matched part (= BLAT hit) for each query, except in cases where queries are found on multiple non-overlapping targets (contigs).

At the end of the output, a list of the unmatched sequences is shown.

  

#### Description of keys

##### Keys for BLAT hits

Every BLAT hit is described by the following keys:

|  |  |
| --- | --- |
| ID | The id of the BLAT hit (equals the line number in the psl file). |
| status | One of "complete", "partial", "incomplete" or "manual". "complete" means that Scipio had no problems locating the query, "partial" means that the hit is on one of multiple targets each matching a part of the query, "incomplete" means there might be the need to edit it manually. "manual" can be entered if the output was modified by hand. |
| reason | If status is "incomplete", the reason why. |
| prot\_len | The length of the query (in amino acid coordinates). |
| prot\_start | Start of the matched part of the query if larger than zero. |
| prot\_end | End of the matched part of the query if less than prot\_len. |
| prot\_seq | The query sequence (in a partial hit: only the matched part). |
| target | The name of the target sequence. |
| target\_len | The total length of the target sequence. |
| strand | "+" (= forward) or "-" (= reverse). |
| dna\_start | The location of the hit. |
| dna\_end |  |
| matches | The number of matches if less than prot\_len. |
| mismatches | The number of mismatches. |
| undetermined | The number of undetermined residues. |
| unmatched | The number of unmatched query residues. |
| additional | The number of additional residues in the translated target. |
| score | The score of the hit. |
| upstream | DNA Sequence upstream of hit, ending before start codon. |
| upstream\_gap | (unaligned) DNA Sequence preceding a partial hit. |
| matchings | The locations of exons and introns, see next section. |
| stopcodon | Stop codon if present. |
| downstream | DNA Sequence downstream of hit, starting with stop codon if present. |
| downstream\_gap | (unaligned) DNA Sequence following a partial hit. |

The commandline parameter --show=*〈comma-separated list〉* can be used to choose a user-defined
collection of keys to be shown. By default, all of the above are shown.

  

##### Keys for matchings

Every matching (intron/exon) is given with the following keys:

|  |  |
| --- | --- |
| type | "intron","exon" or "gap". |
| nucl\_start | Location in the query (in nucleotide coordinates; nucl\_end not for introns). |
| [nucl\_end] |
| dna\_start | Location in the target. |
| dna\_end |
| seq | DNA sequence of the feature. |

  
Keys that appear only in exons:

| seqshifts | Location of sequence shifts. |
| mismatchlist | Positions of mismatches (in query coordinates). |
| undeterminedlist | Positions of undetermined residues (in query coordinates). |
| inframe\_stopcodons | Positions of in-frame stopcodons (in query coordinates). |
| translation | Translation of the aligned part of the DNA sequence. |
| overlap | Number of nucleotides at the end of the target that are identical with the beginning of the following target, and not considered part of this location. |

The commandline parameters --show\_exon=〈...〉, --show\_intron=〈...〉, --show\_gap=〈...〉 can be used to choose user-defined output for matchings.
The following additional keys can be activated by this:

| nucl\_pos | In introns, a synonym for nucl\_start(=nucl\_end)| prot\_start | The location transformed into residue coordinates rather than nucleotides. A remainder of 1 is rounded down, and 2 is rounded up. | | prot\_end | | prot\_pos | (this one for introns) | | prot\_seq | Part of the query matching an exon, or unmatched part in a gap. | |

  

#### Sequence coordinate conventions

All nucleotide and residue coordinates specify the number of preceding letters, that
is, unlike in GFF, a location specified as starting at 5 and ending at 9 has length four,
starting with the sixth and ending with the ninth character. (GFF would show this as
start=6 end=9).
